# Intramuscular Immunisation with Chlamydial Proteins Induces *Chlamydia trachomatis* Specific Ocular Antibodies

**DOI:** 10.1371/journal.pone.0141209

**Published:** 2015-10-26

**Authors:** Alexander Badamchi-Zadeh, Paul F. McKay, Martin J. Holland, Wayne Paes, Andrzej Brzozowski, Charles Lacey, Frank Follmann, John S. Tregoning, Robin J. Shattock

**Affiliations:** 1 Mucosal Infection & Immunity Group, Section of Virology, Imperial College London, St Mary’s Campus, London, United Kingdom; 2 London School of Hygiene and Tropical Medicine, Keppel St, London, United Kingdom; 3 Centre for Immunology and Infection, Hull York Medical School, University of York, York, United Kingdom; 4 York Structural Biology Laboratory, Department of Chemistry, University of York, York, United Kingdom; 5 Chlamydia Vaccine Research, Department of Infectious Disease Immunology, Statens Serum Institut, Copenhagen, Denmark; Univ. of Texas Health Science Center at San Antonio, UNITED STATES

## Abstract

**Background:**

Ocular infection with *Chlamydia trachomatis* can cause trachoma, which is the leading cause of blindness due to infection worldwide. Despite the large-scale implementation of trachoma control programmes in the majority of countries where trachoma is endemic, there remains a need for a vaccine. Since *C*. *trachomatis* infects the conjunctival epithelium and stimulates an immune response in the associated lymphoid tissue, vaccine regimens that enhance local antibody responses could be advantageous. In experimental infections of non-human primates (NHPs), antibody specificity to *C*. *trachomatis* antigens was found to change over the course of ocular infection. The appearance of major outer membrane protein (MOMP) specific antibodies correlated with a reduction in ocular chlamydial burden, while subsequent generation of antibodies specific for PmpD and Pgp3 correlated with *C*. *trachomatis* eradication.

**Methods:**

We used a range of heterologous prime-boost vaccinations with DNA, Adenovirus, modified vaccinia Ankara (MVA) and protein vaccines based on the major outer membrane protein (MOMP) as an antigen, and investigated the effect of vaccine route, antigen and regimen on the induction of anti-chlamydial antibodies detectable in the ocular lavage fluid of mice.

**Results:**

Three intramuscular vaccinations with recombinant protein adjuvanted with MF59 induced significantly greater levels of anti-MOMP ocular antibodies than the other regimens tested. Intranasal delivery of vaccines induced less IgG antibody in the eye than intramuscular delivery. The inclusion of the antigens PmpD and Pgp3, singly or in combination, induced ocular antigen-specific IgG antibodies, although the anti-PmpD antibody response was consistently lower and attenuated by combination with other antigens.

**Conclusions:**

If translatable to NHPs and/or humans, this investigation of the murine *C*. *trachomatis* specific ocular antibody response following vaccination provides a potential mouse model for the rapid and high throughput evaluation of future trachoma vaccines.

## Introduction

Trachoma is a chronic disease caused by the infection of conjunctival epithelial cells with *Chlamydia trachomatis*. It is the world’s leading cause of preventable blindness, with almost 8 million people visually impaired by trachoma [1]. Infection of the conjunctiva is most frequently restricted to serovars A—C of *C*. *trachomatis*, with the D-K serovars causing genital infections. At least 232 million people live in current trachoma endemic areas and are at risk from the disease. Trachoma is currently the target of a global campaign to eliminate disease as a public health problem by 2020 [2]. A vaccine against *C*. *trachomatis* would provide a more definitive solution that targets interruption of transmission and would greatly in aid the long term and irreversible control of trachoma.

Research into the protective immune responses required to prevent trachoma is currently on-going, using both non-human primate (NHP) models alongside immunoepidemiological studies in trachoma endemic populations. In NHPs, Kari *et al*. recently investigated some of the immunological correlates of the protracted clearance of a primary *C*. *trachomatis* ocular infection [3]. In these experiments they infected NHPs with a recent, virulent ocular clinical isolate of *C*. *trachomatis* (A2497). The sera from infected NHP recognised the antigenically variable major outer membrane protein (MOMP) and a few antigenically conserved antigens (PmpD, Hsp60, CPAF and Pgp3). The humoral response to the different antigens had distinct kinetics. Recognition of MOMP occurred rapidly (2–4 weeks) post-infection, and correlated with a reduction in infectious ocular burdens, but not with infection eradication. Antibodies specific to the conserved antigens PmpD, Hsp60, CPAF and Pgp3 appeared later (9–14 weeks post infection) and these antibodies correlated with clearance of infection. MOMP is highly variable and the determinant of serotype classification. It comprises up to 60% of the total elementary body (EB) protein and is a transmembrane protein with a potential porin function [4, 5]. Polymorphic membrane protein D (PmpD) is an outer membrane protein found on the surface of chlamydial EBs and has previously been shown to generate pan-neutralising antibodies [6]. PmpD is highly conserved and involved in chlamydial attachment to host cells [7, 8]. Pgp3 is the product of a highly conserved gene contained within the episomal cryptic plasmid of *C*. *trachomatis* that is secreted into the inclusion lumen and the host cell cytosol. Purified Pgp3 can stimulate macrophages to release inflammatory cytokines suggesting a role in chlamydial pathogenesis [9].

As trachoma is an ocular disease and MOMP, PmpD, and Pgp3 specific antibodies correlated with reduction and eventual clearance, we investigated a number of vaccination regimens to induce ocular antibodies specific to these potentially important chlamydial antigens. A screen of prime-boost regimens using vaccines expressing MOMP revealed which regimens were capable of inducing the highest concentrations of anti-MOMP antibodies on the murine eye. This regimen was then used with the additional recombinant protein antigens PmpD and Pgp3 to investigate whether antigen specific antibodies could be raised against all components of a vaccine cocktail. By exploring chlamydial antigen-specific ocular antibodies elicited following immunisation in mice this study provides a screening platform for future trachoma vaccines.

## Materials and Methods

### Ethics Statement

All animals were handled, procedures performed and the study carried out in strict accordance with the conditions of a project licence granted under the UK Home Office Animals (Scientific Procedures) Act 1986. The protocol was peer reviewed and approved by the Imperial College Ethical Review Process (ERP) Committee and any amendments were peer-reviewed and approved by the Imperial College Animal Welfare and Ethical Review Body (AWERB). Animals received minimal handling and procedures were performed under isoflurane anesthesia when appropriate. Food and water were supplied ad libitum. Animals were monitored and assessed for health and condition daily. No animals became severely ill or died at anytime prior to the experimental endpoint. The method of euthanasia for all animals in this research was cervical dislocation.

### Plasmid, viral vectors and recombinant proteins

Codon optimised MOMP DNA from *C*. *trachomatis* E-Bour was synthesised by GeneArt (Invitrogen, UK) and cloned into pcDNA3.1 (Invitrogen, UK). MOMP was recombined by homologous recombination into the E1 and E3 deleted HuAd5 genome plasmid, pAL1112, kindly provided by Prof Gavin Wilkinson, Cardiff University. MOMP was recombined into the MVA pox vector by the Viral Vector Core Facility, The Jenner Institute (Oxford, UK). Recombinant MOMP cloned from *C*. *trachomatis* serovar D/UW3/Cx expressed in BL21 (DE3) *E*. *coli* was provided by Dr Frank Follmann, Statens Serum Institut (Copenhagen, Denmark). Recombinant PmpD 65kDa passenger domain fragment (aa68-aa698) cloned from *C*. *trachomatis* serovar E-Bour DNA (ATCC) expressed in BL21 (DE3) *E*. *coli* was provided by Prof. Andrzej Marek Brzozowski, University of York. The *C*. *trachomatis* serovar D *pgp3*-GST expression plasmid was provided by Dr Guangming Zhong, University of Texas Health Science Center at San Antonio, and the protein produced as described by Li previously [10]. Recombinant proteins were formulated with the adjuvant MF59® (Novartis) for intramuscular immunisations and with the adjuvant monophosphoryl Lipid A (MPLA, Invivogen) for intranasal immunisations.

### Mice, immunization and sampling

Female 6–8 weeks old BALB/c mice (Harlan, Christ Church, UK) received immunisations at three-week intervals. DNA vaccinations were at 10 μg doses, intramuscularly into the hind quadriceps muscle in a volume of 50 μl with electroporation. Electroporation was with 5 mm electrodes at the immunisation site using an ECM 830 Square Wave Electroporation System (BTX), with three pulses of 100 V each, followed by three pulses of the opposite polarity with each pulse (P_ON_) lasting 50 ms and an interpulse (P_OFF_) interval of 50 ms. All HuAd5 and MVA vaccinations were at dosages of 10^7^ PFU and 10^6^ PFU respectively. rMOMP was administered at a dose of 10 μg, rPmpD at 5 μg and rPgp3 at 10 μg, in a 1:1 mixture with MF59® in a final volume of 50 μl for intramuscular immunisations. Samples were collected two weeks after each immunisation. Blood was collected and centrifuged at 1,000 g for 10 min. The serum was harvested and stored at -20°C. Vaginal lavage was performed using three 25 μl washes/mouse with sterile phosphate buffered saline (PBS) that were later pooled. Lavage samples were incubated with protease inhibitor (Roche Diagnostics, Germany) before centrifuging at 1,000 g for 10 min. The fluid supernatant from these samples was harvested and stored at -20°C. For ocular secretions 10 μl of ocular extraction buffer (1.5 g sodium chloride, 20 μl of 10% sodium azide solution in 100 ml Dulbecco’s Phosphate Buffered Saline (DPBS)) was pipetted onto each eye and subsequently absorbed with 2 mm x 5 mm PVA swabs. These swabs were then incubated with an additional 50 μl of ocular extraction buffer at room temperature before centrifugation at 500 g for 5 min through a 0.45 μm filter. The eluate was harvested and stored at 4°C until used.

### Immunoglobulin semi-quantitative ELISA

A semi-quantitative immunoglobulin ELISA protocol described previously [11] was used. Briefly, 0.5 μg/ ml of either rMOMP, PmpD or Pgp3 coated ELISA plates were blocked with 1% BSA/0.05% Tween-20 in PBS. After washing, diluted samples were added to the plates for 1 hr before repeat washing and the addition of a 1:4,000 dilution of either anti-mouse IgG-HRP or IgA-HRP (Southern Biotech). Standards consisted of coating ELISA plate wells with anti-mouse Kappa (1:3,200) and Lambda (1:3,200) light chain (Serotec, UK), blocking with PBS/1% BSA/0.05% Tween-20, washing and then adding purified IgG or IgA (Southern Biotech, UK) starting at 1,000 ng/ml. Samples and standards were developed using TMB (3,3′,5,5′-Tetramethylbenzidine) and the reaction stopped after 5 min with Stop solution (Insight Biotechnologies, UK). Absorbance was read on a spectrophotometer (VersaMax, Molecular Devices) with SoftMax Pro GxP v5 software.

### Statistical Analysis

All statistical analyses were carried out using Prism 6.0 (GraphPad, USA). The distribution of the data was assessed using the Kolmogorov Smirnov normality test. For non-parametric data the Kruskal-Wallis test with Dunn’s multiple comparison post-test was used to compare more than two groups, or the two-tailed Mann-Whitney test to compare two groups. For parametric data, a one-way ANOVA was used for multiple comparisons, with Tukey’s multiple comparison post-test for comparison of specific groups. p ≤ 0.05 was considered significant (* p ≤ 0.05, ** p ≤ 0.005, *** p ≤ 0.0005 and **** p ≤ 0.0001).

## Results

### Heterologous prime-boost vaccine regimens induce MOMP-specific ocular IgG

**Fig 1:**
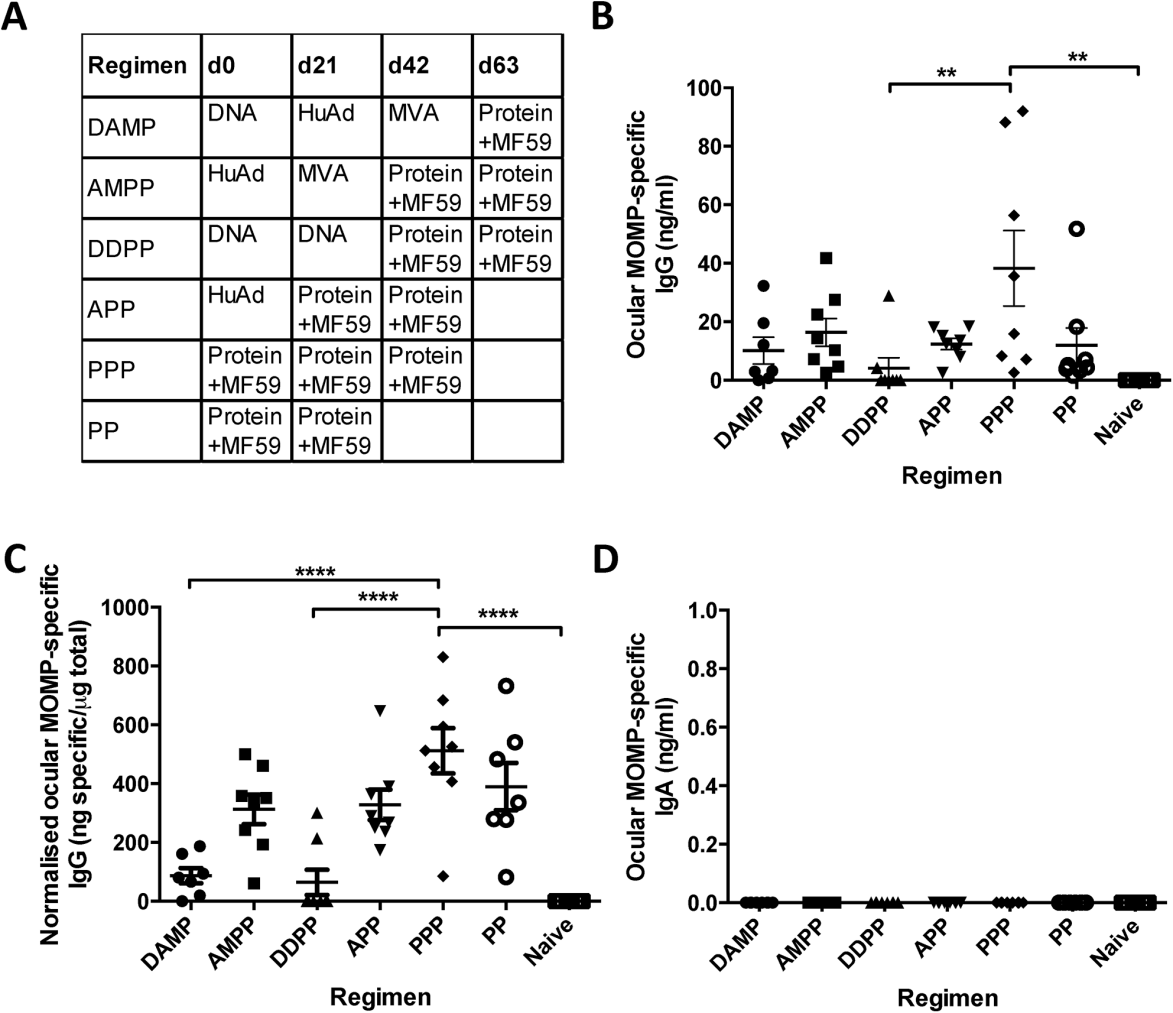
Ocular IgG and IgA responses following heterologous prime-boost intramuscular vaccination. BALB/c mice were immunised using different heterologous prime-boost vaccine regimes (A). Samples from the surface of the eye were collected two weeks after the final immunisation and assessed by ELISA for MOMP-specific ocular IgG (B). Specific IgG was normalised to the level of total ocular IgG in the same sample (C). MOMP specific IgA was in ocular fluid was assessed by ELISA (D). Points represent individual mice, lines represent mean (n = 8 per group), +/- SEM. ** p ≤ 0.005, and **** p ≤ 0.0001 by one-way ANOVA with Tukey’s multiple comparison post-test.

BALB/c mice were immunised intramuscularly (IM) with differing combinations of heterologous prime-boost regimens consisting of DNA (D), HuAd5 (A), MVA (M) and protein (P) MOMP-based vaccines (Fig 1A). The protein immunisations were co-administered with MF59 adjuvant. Mice have previously been shown to not have pre-existing immunity to HuAd5 [12]. Immunisations were administered three weeks apart and the ocular mucosa sampled two weeks after the final immunisation (Fig 1B). The PPP regimen induced significantly higher MOMP-specific ocular IgG than naïve unvaccinated controls (** *p* ≤ 0.005, one-way ANOVA with Tukey’s multiple comparison post-test) or the DDPP group (** *p* ≤ 0.005). PPP + MF59 induced the highest mean MOMP-specific ocular IgG concentrations of 38.28 ng/ml. The DDPP regimen induced the lowest mean MOMP-specific ocular IgG concentrations with 4.13 ng/ml. The proportion of total ocular IgG that was specific to the antigen in each heterologous prime-boost vaccination regimen was calculated by normalising the MOMP-specific ocular IgG concentrations to the total IgG (per 1 μg total IgG) concentrations of the ocular samples (Fig 1C). The PPP regimen induced MOMP-specific ocular IgG antibodies that accounted for more than half of the total IgG in ocular secretions (mean = 511 ng specific/μg total). The AMPP, APP and PP regimens induced MOMP-specific ocular IgG antibodies accounting for around a third of the total IgG in ocular secretions, with DAMP and DDPP inducing less than 10% of the total IgG to be MOMP-specific. No antigen specific IgA was detected in any of the immunised groups (Fig 1D) yet the mean total IgA concentrations ranged from 601–1012 ng/ml between immunised groups.

### Mucosal immunisation boosts IgA response but does not lead to greater ocular responses

**Fig 2:**
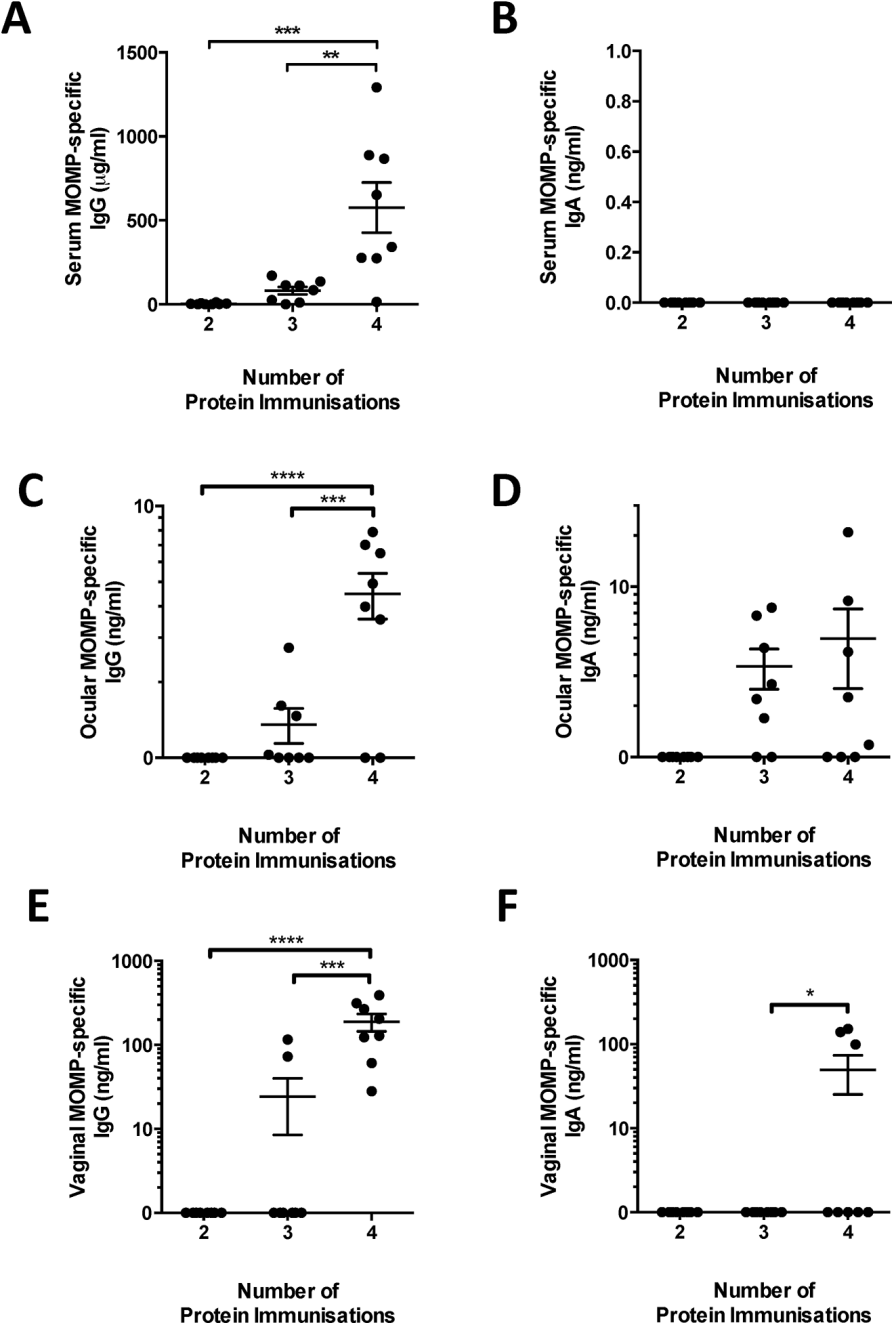
Intranasal immunization with protein and MPLA induces antigen-specific IgA ocular secretion. BALB/c mice were intranasally immunized with MOMP adjuvanted with MPLA. Blood, ocular and vaginal lavages were collected 2 weeks after the 2nd, 3rd and 4th immunizations. MOMP-specific IgG (A, C, E) and IgA (B, D, F) were measured in serum (A, B), ocular lavage (C, D) and vagina lavage (E, F) by ELISA. Points represent individual animals, lines represent means of n = 8 per group +/- SEM. * p ≤ 0.05, ** p ≤ 0.005 and *** p ≤ 0.0005 by one-way ANOVA with Tukey’s multiple comparison post-test.

It has been hypothesized that mucosal sites are linked immunologically [13]. This suggests that mucosal immunisation might lead to increased ocular antibody responses. Mice were immunised intranasally with 10 μg MOMP protein with MPLA (receiving 4 immunisations at 3 week intervals). MPLA was used as it is a well-characterised mucosal adjuvant [14]. Antibody responses were measured in the serum, ocular secretions and in a distal mucosal site–the vagina. There were low levels of detectable MOMP specific serum IgG (Fig 2A) responses in animals after two and three intranasal doses of protein, though levels were not significantly elevated. A significant increase in serum IgG was only observed after four protein immunisations. No serum IgA was detected at any time-point after immunisation (Fig 2B). The ocular antibody response was measured in the same animals. As with serum responses, significant levels of ocular MOMP-specific IgG was only detectable after the 4^th^ protein immunisation, though the absolute level (mean = 4.5 ng/ml) was lower than measured after intramuscular immunisation (mean = 38.3 ng/ml). Strikingly, detectable levels of MOMP specific IgA were measured in some of the animals after 3 and 4 immunisations (Fig 2D). We also measured the MOMP-specific IgG response in the vagina, a mucosal site distal to the route of vaccination. Antigen-specific IgG (Fig 2E) was detectable in all animals while MOMP-specific IgA (Fig 2F) was detectable in 3 out of 7 animals after 4 doses of protein. From this we conclude that intranasal immunisation can induce mucosal responses at remote sites, but it requires more mucosal immunisations than intramuscular to achieve a statistically significant elevated level of response in the eye, and the absolute level was lower than intramuscular immunisation. Interestingly, intranasal immunisation induced a mucosal response but did not induce a systemic IgA response.

### Mucosal delivery of viral vectors fails to induce local responses

**Fig 3:**
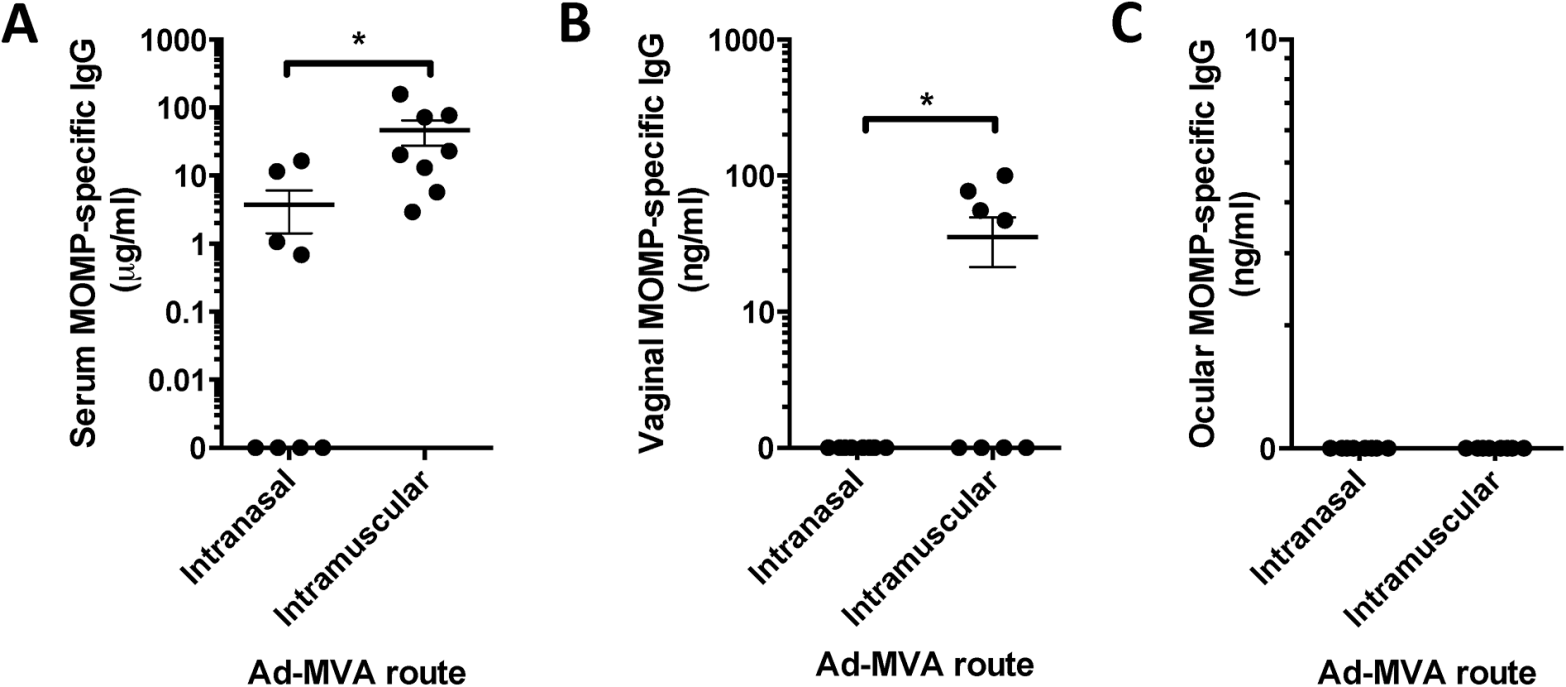
Intranasal immunisation with Adenovirus and MVA vectors failed to induce a mucosal antibody response. BALB/c mice were immunised intranasally or intramuscularly with Adenovirus and then MVA with a 3-week interval. MOMP-specific IgG was measured in serum (A), vaginal lavage (B) and ocular lavage (C). Points represent individual animals, lines represent means of n = 8 per group +/- SEM. * *p* ≤ 0.05 by unpaired t test.

Since mucosal immunisation with a protein antigen induced a local IgA response, we wished to determine whether engineered viral vectors were able to induce a similar response. Viral vectors can infect multiple cell types, including mucosal epithelia, and have been demonstrated to be effective for mucosal immunisation in influenza models [15]. Mice were intranasally (IN) immunised with an adenovirus (HuAd5) expressing MOMP followed by a modified vaccinia Ankara (MVA) expressing MOMP regimen. Local and ocular immune responses were measured and compared to intramuscular (IM) delivery of the vector. IM delivered vectors (Ad Prime: MVA Boost) induced a significantly greater serum (p<0.05, Fig 3A) and vaginal (p<0.05, Fig 3B) IgG response than IN delivery. There was no MOMP-specific IgG antibody response detectable at the ocular surface after vector vaccination by either route (Fig 3C).

### Immunisation generated ocular antibody responses against MOMP and Pgp3 but not PmpD

**Fig 4:**
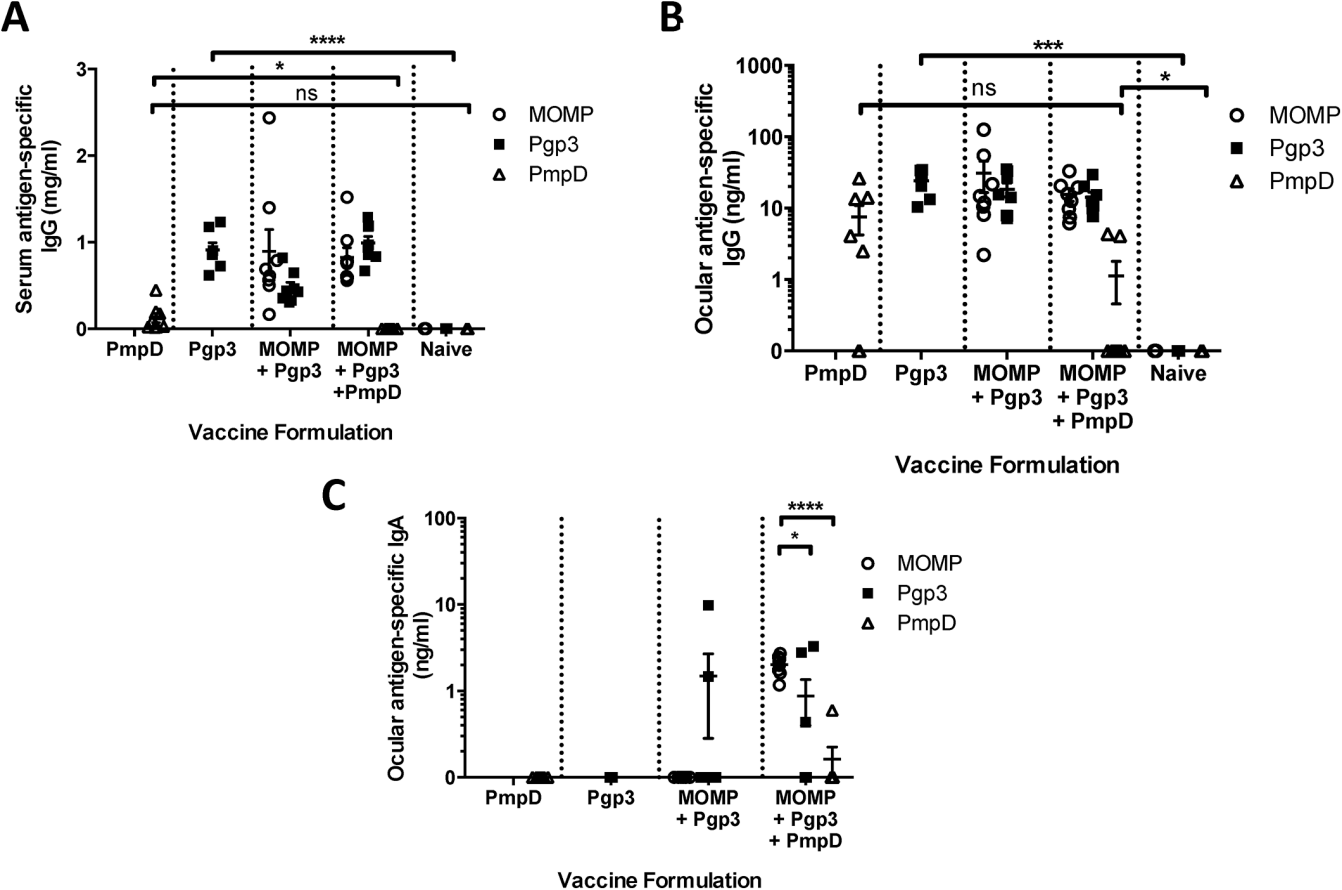
Recombinant protein vaccines adjuvanted with MF59 induced specific ocular IgG antibodies. BALB/c mice were immunised three times intramuscularly either with a single protein or a combination of proteins with MF59. Two weeks after the final immunisation serum (A) and ocular lavage (B, C) were collected. Antigen-specific IgG in serum (A) and ocular antigen-specific IgG (B) and IgA (C) were assessed by ELISA. Points represent individual animals, lines represent means of n = 8 per group +/- SEM. * p ≤ 0.05, ** p ≤ 0.005, *** p ≤ 0.0005 and **** p ≤ 0.0001 by unpaired t test.

Having observed the greatest induction of ocular antibody after intramuscular delivery of MOMP protein adjuvanted with MF59, we wished to determine whether other chlamydial proteins, PmpD and Pgp3, could also induce ocular antibodies after immunisation. PmpD and Pgp3 were chosen because of their implicated role in chlamydial eradication. The additional antigens PmpD and Pgp3 were investigated in cocktail vaccine formulations that might potentially be used in a final vaccine product. Mice were vaccinated intramuscularly with individual proteins or combinations of recombinant proteins adjuvanted with MF59; PmpD alone, Pgp3 alone, MOMP + Pgp3, and MOMP + Pgp3 + PmpD (Fig 4A). Both PmpD and Pgp3 induced an IgG response in the serum after 3 immunisations, with anti-Pgp3 IgG concentrations significantly greater than in naïve animals. Interestingly the specific anti-PmpD serum responses were lower than responses to other antigens and when the PmpD antigen was included in a cocktail with other antigens, the anti-PmpD response in the serum was significantly lower than when administered alone (p<0.05). Combining antigens had no effect on the response to either MOMP or Pgp3.

A similar effect was observed in the ocular IgG responses, with MOMP and Pgp3 inducing comparable anti-IgG responses. Pgp3-specific ocular IgG concentrations were consistent whether immunised with Pgp3 alone (mean = 24.2 ng/ml) or immunised in combination with MOMP (mean = 18.4 ng/ml) or with MOMP and PmpD (mean = 14.2 ng/ml). MOMP-specific ocular IgG concentrations were mean = 31.1 ng/ml when co-administered with Pgp3, and mean = 15.6 ng/ml when co-administered with Pgp3 and PmpD (Fig 4B). As seen in the serum, the combination of PmpD with other antigens reduced the anti-PmpD response, though not significantly. Anti-PmpD ocular IgG concentrations (PmpD alone, mean = 7.5 ng/ml; PmpD with MOMP and Pgp3, mean = 1.06 ng/ml) were lower than MOMP- or Pgp3-specific ocular IgG concentrations. As seen with MOMP alone, the chlamydial antigens Pgp3 and PmpD did not induce antigen-specific ocular IgA antibodies in detectable concentrations alone (Fig 4C). MOMP in combination with Pgp3 also did not induce detectable ocular IgA responses. Interestingly, the combination of MOMP with PmpD and Pgp3 induced ocular IgA responses against both MOMP and Pgp3, which was not seen when they were used alone or in combination without PmpD. The number of animals responding to Pgp3 was also increased, but there was no effect on the anti-PmpD response (MOMP vs Pgp3, p = 0.0394; MOMP vs PmpD, p < 0.0001).

## Discussion

We screened heterologous prime-boost vaccination regimens using DNA, HuAd5, MVA and adjuvanted protein vaccines to reveal which regimens are capable of inducing *C*. *trachomatis* antigen-specific antibodies in ocular secretions. To the best of our knowledge, our sampling of *C*. *trachomatis*-specific antibodies in ocular secretions in the mouse following vaccination is unique, with ocular antibodies previously only sampled from non-human primates (NHPs) [16–18] or humans [19, 20]. Three administrations of protein adjuvanted with MF59 induced the highest MOMP-specific ocular IgG concentrations. Not all *C*. *trachomatis* antigens were capable of inducing equal concentrations of antigen-specific IgG. PmpD induced less antigen-specific IgG than either Pgp3 or MOMP antigens both at the ocular mucosal surface and systemically. Four intranasal administrations of MOMP adjuvanted with MPLA induced antigen-specific IgA antibodies in ocular secretions, though it led to lower systemic and ocular IgG responses. Kari *et al*. showed that specific serum antibody signatures correlated with reduction in ocular *C*. *trachomatis* load and differing signatures correlated with clearance of ocular infection in macaques [3]. Our data reveals a vaccination regimen to induce ocular anti-MOMP and anti-Pgp3 responses on the murine eye, allowing the future prospect of screening the specific antibody induction potential of new *C*. *trachomatis* vaccine antigens.

Though there is a high concentration of measurable total IgA on the murine ocular mucosa, systemic immunisation with MOMP, PmpD, or Pgp3 proteins did not induce antigen specific IgA when delivered individually. This could be due to the antigen, the adjuvant or the intramuscular route of immunisation. We show that four intranasal protein MOMP immunisations adjuvanted with MPLA are capable of inducing MOMP-specific IgA, both ocularly and vaginally, but interestingly not with vectored vaccines. It would be of interest to investigate whether alternative adjuvants in the protein regimen elicit equally high IgG concentrations but of differing isotypes, as MF59 skews the IgG type to IgG1 predominance [21, 22], which may alter levels at the ocular mucosal surface. Likewise, just as experimental mucosal adjuvants can boost the IgA response [23, 24] using the same antigen, different adjuvants can influence the predominant antibody isotype in mice [14, 25]. However, further studies are required to confirm these findings in higher animals as we have recently observed that mucosal adjuvants that are effective in mice are less effective in non human primates [26]. It has been suggested that local stimulation recruits T cells to mucosal surfaces after systemic immunisation [27] but we have recently observed that this is not effective for B cell responses [28] and there may be an unacceptable risk of applying inflammatory substances to the ocular mucosal surface. It was of note that combining the antigens increased the ocular IgA response, it is unclear why the combination was better able to elicit anti-MOMP IgA but may reflect more immune activation following exposure to multiple antigens. But this effect was variable, with the addition of MOMP and Pgp3 to PmpD suppressing the anti-PmpD response.

There is a school of thought that antibodies do not have a role in controlling trachoma. Bailey *et al*., found that the presence of anti-chlamydial IgG antibodies in ocular secretions of disease-free individuals was in fact associated with an increased incidence of trachoma. This led the authors to conclude that anti-chlamydial IgG, but not IgA, antibodies could possibly enhance the infectivity of *C*. *trachomatis* [29, 30]. Both the IgG isotype and the antigen targeted might be of importance, as isotype differences and antigen-specificities have shown differential uptake and translocation of *Chlamydia* sp. into cells *in vitro* [31]. Kari *et al*. have previously shown no correlation between anti-chlamydial sera or tear IgG and IgA titres and protective immunity, though the authors are cautious to note that their ELISAs used intact elementary bodies as the test antigen and were thus only able to detect a very limited number of surface proteins [32]. Antibodies may not be effective in natural infection, but vaccine induced protection can act through different mechanisms to that of naturally acquired immunity. Olsen *et al*. have recently shown that neutralising antibodies specific for the VD4 region of MOMP can protect upon vaginal infection if present at high titres [33], and previous findings that protective immunity against *C*. *trachomatis* ocular infection has no cross-protection against different serovars indirectly implicate serovar-specific neutralising antibodies in ocular immunity [34]. Our PPP regimen with the MOMP protein adjuvanted with MF59 has induced sera with neutralising ability against *C*. *trachomatis* EBs (Badamchi-Zadeh *et al*. (2015) In submission), suggestive that the antibodies could potentially be protective. Nevertheless, the significance of antibodies in ocular protection needs to be further defined. The observed antibody signatures in NHP and human studies may simply be surrogate markers of protective T cell immunity, which has recently further been implicated in genital protection [35].

We have developed a novel model for the sampling and analysis of ocular antibodies in mice and demonstrate that vaccination regimens can be configured to induce significant concentrations of *C*. *trachomatis* antigen-specific antibodies on the ocular mucosa. Although the proteins used in our immunisations were derived from *C*. *trachomatis* serovars D or E, less commonly associated with trachoma, this provides proof of concept for induction of ocular responses. Further studies will be needed to assess responses using matched proteins from serovars A-C, the predominant etiological agents of trachoma. A second limitation to our study is that mice, although susceptible to ocular infection [36], are not the standard model for ocular *C*. *trachomatis* pathology, where the use of Guinea pigs [37] or non-human primates are seen as standard models of trachoma [10]. We would predict that the vaccine strategies developed in this study would likely translate to these different models and would allow for the rapid and high throughput evaluation of different trachoma vaccine constructs with subsequent evaluation in humans. Although further work is required to test this experimentally, these new tools may prove insightful in future studies to determine the potential role of antibodies (whether neutralising, opsonising, antibody-dependent cell-mediated cytotoxicity) in protection against trachoma and the development of vaccines against chlamydial induced disease.
